# Anticipation in Lynch Syndrome: Where We Are Where We Go

**DOI:** 10.2174/138920211797904070

**Published:** 2011-11

**Authors:** Cristina Bozzao, Patrizia Lastella, Alessandro Stella

**Affiliations:** Medical Genetics Unit, Department of Biomedicine in Childhood, Università degli Studi di Bari “Aldo Moro”, Bari, Italy

**Keywords:** Anticipation, lynch syndrome, MMR genes, microsatellite instability, telomere length, telomere dynamics.

## Abstract

Lynch syndrome (LS) is the most common form of inherited predisposition to develop cancer mainly in the colon and endometrium but also in other organ sites. Germline mutations in DNA mismatch repair (MMR) gene cause the transmission of the syndrome in an autosomal dominant manner. The management of LS patients is complicated by the large variation in age at cancer diagnosis which requires these patients to be enrolled in surveillance protocol starting as early as in their second decade of life. Several environmental and genetic factors have been proposed to explain this phenotypic heterogeneity, but the molecular mechanisms remain unknown. Although the presence of genetic anticipation in Lynch syndrome has been suspected since 15 years, only recently the phenomenon has been increasingly reported to be present in different cancer genetic syndromes including LS. While the biological basis of earlier cancer onset in successive generations remains poorly known, recent findings point to telomere dynamics as a mechanism significantly contributing to genetic anticipation in Lynch syndrome and in other familial cancers. In this review, we summarize the clinical and molecular features of Lynch syndrome, with a particular focus on the latest studies that have investigated the molecular mechanisms of genetic anticipation.

## INTRODUCTION

Lynch syndrome (LS), also known as hereditary non-polyposis colorectal cancer (HNPCC), is a cancer predisposing syndrome characterized by autosomal dominant inheritance of a germline mutation in the DNA mismatch repair genes. This syndrome represents the most common hereditary form of colorectal cancer (CRC), accounting for 2-4% of the annual worldwide incidence [1]. Individuals with LS develop tumors at an early age, often <45 years. The vast majority of the mutations is highly penetrant, therefore multiple generations in the same family are affected by cancer and skipping of a generation is a rare event [2]. Individuals affected by LS are also predisposed to various types of extracolonic cancers mainly in the endometrium, stomach, ovary, hepatobiliary tract, upper urinary tract, small bowel, brain and pancreas [3]. There are conflicting evidences of a higher risk of breast and prostate cancers associated with the syndrome [4,5]. The lifetime risk of cancer development depends on patients’ sex and the MMR-gene involved. In mutation carriers, the lifetime CRC risk is estimated to be 50-80% [6]. In women the higher risk is for endometrial cancer, which is estimated to be 40-60%, with LS being diagnosed in approximately 2% of all endometrial cancer patients [7]. The risk for other extra-colonic cancers associated with MMR genes mutations is cumulatively less than 10% [8], and these cancers are more frequently associated to MSH2 mutations [9,10]. In LS, CRCs are typically located in the proximal colon (two-thirds of cases) and patients present a high frequency of multiple tumors, either synchronous or metachronous (35% of patients) [2]. LS tumors are also less likely to metastasize than sporadic ones [11,12].

## MMR GENES, MSI AND TUMORIGENESIS

Most of the disease causing mutations in LS fall in one of four genes all involved in the DNA-mismatch repair system (MMR): MSH2, MLH1, MSH6 and PMS2 [13]. MMR genes encode for proteins involved in the identification and repair of replication errors, such as mispaired nucleotides or small insertion/deletion loops (IDLs), caused by the slippage of DNA polymerase while replicating DNA regions containing short tandem repeats (STRs) also known as microsatellite loci. In eukaryotes the mismatch repair requires the presence of two heterodimeric complexes called MutS and MutL. In mammalian cells, the MutS heterodimer recognizes replication errors in the form of mismatches, and recruits MutL to the site of damage. MutSα, formed by MSH2-MSH6, recognizes single base mismatches or insertion/deletion loops, while the MutSβ complex, made by MSH2-MSH3, is responsible for the recognition of IDLs of 2 to 8 nucleotides. The MutLα heterodimer, consisting of MLH1 and PMS2, forms a ternary complex with one of the MutS complexes bound to the mismatch site, and together with ExoI, PCNA (Proliferating Cell Nuclear Antigen) and DNA polymerase, leads to strand discrimination, error removal and DNA resynthesis [14]. 

So far, LS has been associated with mutations in seven different genes: MLH1, MSH2, MSH6, PMS1, PMS2, MLH3 and EXO1 [15]. More recently, germline deletions of the EpCAM gene (epithelial cell adhesion molecule, formerly known as TACSTD1) have been identified in several LS families/cases and are recognized as a novel class of mutations predisposing to LS [16]. Despite the increasing sensitivity of mutation screening techniques in candidate genes, in a subset of families fulfilling Amsterdam I or II criteria (40-70%) an MMR gene defect has not been identified yet [17,18]. These families have been referred since then as familial colorectal cancer *type X* [19], and are characterized by MSS tumors, lower cancer risk and later onset of CRC (10 years after the median age of onset in LS), and a minor risk of extracolonic cancers compared to both MSH2 and MSH6 mutation carriers. The molecular genetics of familial colorectal cancer *type X* is still unknown but new techniques, such as Next Generation Sequencing (NGS) technology, appear to be a promising tool in identifying gene mutations and/or a combination of genetic variants associated with cancer predisposition in this subgroup of families. In fact, the possibility to rapidly produce an enormous volume of data cheaply, makes feasible the simultaneous screening of several candidate genes including regions non-traditionally target of mutations (such as introns and untranslated regions). Novel specialized platforms based on NGS are apparently ready to hit the market and to revolutionize MMR genes mutation screening, as it was the case for Multiplex Ligation Probe Amplification (MLPA).

Indeed, MLPA analysis turned out to be extremely useful in identifying genomic rearrangements in MMR genes which account for nearly 30% of all germline mutations identified so far, including germline deletions in the EpCAM gene. In LS patients harboring a germline mutation, somatic loss or inactivation of the wild-type allele, through point mutations, methylation, gene conversion or LOH, results in a generalized genomic instability recognized as the accumulation, with high frequency, of mutations in these STR sequences known as microsatellite instability (MSI). MSI is the hallmark of LS and the presence of high levels of instability (MSI-H) in a tumor tissue proves the association of cancer with the syndrome. As a consequence, MSI testing is highly recommended whenever a patient is suspected of having LS.

In 1997, the National Cancer Institute proposed a panel of 5 microsatellites, known as the Bethesda panel [20], to test the MSI status and classify tumors in three main categories: tumors that do not show MSI termed microsatellite stable (MSS); tumors showing a low level of instability (MSI-L which is generally considered the presence of single altered microsatellite marker in the Bethesda panel of five loci or up to two in an extended panel of ten); and tumors MSI-H with instability in more than 20% of the microsatellite marker loci analyzed. MSI-L tumors, with instability mostly limited to mononucleotides repeats, have been frequently but not exclusively associated with mutations in the MSH6 gene [21,22]. In many cases, tumors with low-level of microsatellite instability are biologically and histologically similar to the MSS ones although the use of expanded panel of mononucleotide markers has been recently demonstrated effective in reclassifying the majority of MSI-L cases as either MSI-H or MSS [23-27]. Because mononucleotide markers allow a more accurate assessment of MSI [28,29], using these markers the sensitivity and specificity for detection of MSI reached nearly 100% [29]. While the MSS category is largely prevalent in sporadic cancers, the MSI-H status has been identified in up to 15-20% of all CRCs, most of which are sporadic. A genetic predisposition causing high levels of instability has been identified only in 1-4% of MSI-H CRCs and after the identification of a germline mutation in one of the MMR genes the Lynch syndrome is diagnosed. In sporadic MSI-H tumors the presence of instability is generally caused by the silencing of the MLH1 gene through the somatic hypermethylation of its promoter, which leads to the complete loss of the MLH1 protein. The presence of the BRAF V600E mutation in MSI-H, MLH1-negative CRCs, is a highly specific marker for the sporadic origin of the cancer [30,31]. On the contrary, mutations in the beta-catenin, mainly in exon 3, have been observed only in MSI-H tumor of patients with LS [32].

The “mutator phenotype”, caused by the inactivation of the MMR system, is also characterized by the accumulation of mutations in different target genes containing homopolymeric runs of a single nucleotide. The first of these target genes was identified almost two decades ago when, in 90% of the MSI-H CRCs analyzed, a mutation in a stretch of ten consecutive As in the coding sequence of TGFbR2 was found [33]. Since then, many other target genes sharing the presence of short repeat regions in their coding sequence, have been identified and all of them are inactivated by frameshift mutations in a context of MMR deficiency [34]. Among these MSI target genes the large majority encodes for proteins active in pathways of fundamental importance for cell homeostasis such as cell proliferation, cell signaling, cell cycle, apoptosis and DNA repair. Mutations in these genes usually confer a selectable growth advantage to the cells driving the consequent malignant transformation [35]. Interestingly, all human MMR genes, except MLH1, present a polyA repeat in their own coding sequence suggesting that MMR system could gradually lose other components in a context of wide genomic instability. However, the identity of the mutated genes with a functional role in the tumor progression and those that simply represent bystander markers of MSI is not clear yet [36]. 

Although many evidences support the concept that instability caused by MMR deficiency plays a causative role in cancer development, it is likely that the microenvironment of the colon and other cancer sites may contribute to tumor progression and the eventual metastatic process [37].

In Lynch patients the adenoma-carcinoma sequence is accelerated compared to sporadic CRCs. In fact, the progression from adenoma to carcinoma in these patients may take less than 2-3 years compared to 8-10 years observed in sporadic CRC [38]. Adenomas identified in Lynch syndrome patients show peculiar histological features, including a high degree of dysplasia and villous architecture, which are normally related with a more pronounced risk of malignant degeneration. Considering the histologic features and the accelerated progression one would expect a worse prognosis for Lynch patients’ tumors, which is not the case. In contrast, MSI tumors (both LS-related and the MSI-H sporadic) are characterized by a better prognosis compared to MSS sporadic tumors, when controlled for age and stage [39]. Similarly, LS-associated tumors, are also histologically well defined: colorectal cancers are diploid, poorly differentiated, mucinous and present a higher frequency of Crohn’s like reaction and tumor-infiltrating lymphocytes [40]. As previously mentioned, while all these features are usually indicative of a worse prognosis, Lynch patients do survive longer than patients affected by sporadic CRC [41]. In one of the largest studies performed so far on healthy mutation carriers, intensive surveillance and satisfying follow-up compliance contributed to unincreased mortality despite an observed higher cancer risk when compared to non carriers [42]. Thus, the increased surveillance and the intrinsic biological features of LS tumors combine to reduce cancer-related morbidity and mortality [12]. These findings in combination with the presence of multiple primary extracolonic tumors, and with the observed lower frequency of metastases, led to the hypothesis that Lynch patients develop significant defenses against cancer through an enhanced immune response [43]. Both the infiltration of T lymphocytes (TILs) in the tumor and the presence of genomic instability that could reduce tumor vitality have been proposed to explain the better prognosis of LS-associated tumors [44]. The lack of mismatch repair has been shown to generate in MSI-H tumors new immunogenic frameshift peptides (FSPs) that elicit an anti-tumor inflammatory response mediated by CD8+ T cells able to kill tumor cells through a cytotoxic mechanism [45]. Although β2-microglobulin (β2M) mutations leading to the loss of MHC class I antigen presentation to CD8+ T cells have been documented in LS patients [46], no grade IV metastatic MSI-H tumors presented mutations in this gene suggesting a crucial role for β2M in the metastatic process [47]. At the same time the lack of MHC class I expression could trigger a NK cell-mediated killing of these tumor cells, representing another mechanism of tumor control [43].

The prognostic significance of MMR deficiency has been evaluated also in correlation with the response to adjuvant chemotherapy. MMR defective cells have been shown to be more resistant to 5-fluorouracil (5-FU) activity than wild type MMR cells, indicating that MSI tumors may be less sensitive to some chemotherapeutics [48]. Moreover, when MMR deficiency is caused by MLH1 hypermethylation, demethylating its promoter region restores cell sensitivity to 5-FU-induced DNA damage [49]. The majority of the studies performed so far are concordant in reporting that patients with MMR-deficient tumors do not benefit from adjuvant chemotherapy [50-52]. These data led to the conclusion that there are no scientific evidences justifying the treatment of LS patients with adjuvant chemotherapy protocols based on 5-FU [53].

## ASCERTAINMENT CRITERIA, DIAGNOSIS AND FOLLOW UP

Personal and family informations are crucial in identifying patients with a higher risk of CRC. The first criteria proposed to identify LS families were established during a meeting of the International Collaborative group on HNPCC held in Amsterdam in 1991 and then named Amsterdam criteria I. However they fail to detect up to 50% of families with LS [54]. Few years later, the Amsterdam criteria II were revised to include the extra-colonic cancers that are integral part of the syndrome [55]. The Bethesda guidelines originally proposed in 1997 [56], and revised (RBG) in 2004 [57], aimed to incorporate MSI and immunohistochemistry (IHC) testing to improve the identification of cases with delayed onset and/or weak family history. The presence of a young age at first cancer onset in association with a positive family history and/or multiple primary LS-related tumors has been shown effective in selecting the patients who should undergo MSI testing [12]. The presence, in LS-related tumors, of TILs and of the specific histopathological features previously mentioned, are also useful for patient selection [58]. However, the ascertainment of cases based exclusively on family history may miss a substantial proportion of patients carrying germline mutations in MMR genes due to small nuclear families and incomplete anamnesis [59]. To avoid this recruitment bias, more recent approaches recommended including all patients <50 years in presence of CRC or of any other LS spectrum tumor, even in absence of a cancer family history [12]. Because of their high sensitivity and specificity in identifying LS-related tumors, IHC and MSI have been proposed as large-scale first tier screening tests for all patients who develop a CRC [1]. However, the only direct confirmation of LS diagnosis is the identification of a causative mutation in one of the MMR genes. Indeed, although molecular screening of these genes is costly and time consuming it becomes cost-effective when supported by IHC and MSI-based selection.

The MSI phenotype is considered the most effective tool in identifying LS patients and represents a strong prognostic and predictive marker for patients’ clinical management. Considering that 90% of Lynch associated tumors are MSI-H, mutation testing in presence of a positive MSI test in young cancer patients is recommended. 

IHC to detect MLH1, MSH2, MSH6 and PMS2 expression in tumor tissue is routinely used by pathology laboratories to identify a defective MMR system. While the complete loss of expression of MLH1 and MSH2 causes the biallelic inactivation of the corresponding MMR gene, the lack of PMS2 or MSH6 protein expression can also be caused by the instability of the heterodimeric complexes consequent to the loss of MLH1 and MSH2, respectively. In fact, when one of these two proteins is lost, the MutS and MutL heterodimers cannot be assembled leading to the rapid degradation of the corresponding partners, MSH6 and PMS2. In contrast, germline mutations in MSH6 or PMS2 cause isolated loss of the encoded protein in the tumor tissue [60]. The sensitivity of IHC analysis (95%) is comparable to that of MSI analysis and indicates which MMR gene should undergo mutation search. Inaccurate sampling and heterogeneous staining represent major limits of IHC, requiring the supervision of an expert pathologist for a correct diagnosis [61]. Occasionally, cases presenting incomplete loss of the MLH1 protein expression have been reported in patients with a proven germline missense mutation of unknown clinical significance [62]. These mutations may produce a non functional protein still able to be expressed at normal levels. In these cases, the diagnosis of LS should be supported by functional assays of the mutant MMR protein [63]. 

The young age at cancer onset and the relative high incidence of CRC observed in Lynch mutation carriers argue for their tight surveillance. In a recent study, the survival rate at 5 years in patients regularly screened by colonoscopy was nearly 98%, compared with 12% of those who declined surveillance, and the CRC incidence decreased from 27% to 11% [64]. Shorter colonoscopy intervals (1-2 years) and the beginning of the surveillance at a younger age (20-25 years) are helpful in reducing CRC risk and death in mutation carriers. The recently developed chromoscopic colonoscopy appears to improve the detection of suspicious lesions such as flat and pedunculated adenomas compared to standard colonoscopy [65]. Subtotal colectomy, followed by annual screening of the remaining rectal segment, is usually proposed to patients as an alternative to lifetime colonoscopy. Prophylactic surgery is an option for patients with a low compliance for invasive screening procedures [39]. The risk for other LS-related cancers could be also substantially reduced by intensive surveillance and follow-up strategies [66]. The organ-specific surveillance includes transvaginal ultrasound for endometrial cancers, possibly combined with endometrial sampling, starting at the age of 30-35 years. Prophylactic hysterectomy and oophorectomy could be considered when childbearing is completed [67]. In case of family history for gastric and small bowel tumors a periodic upper endoscopy is recommended. Finally, in families with ureter/renal pelvis cancer, patients should have annual ultrasound and urinalysis with cytological examination from age 30 or at first evidence of hematuria [53].

## GENOTYPE-PHENOTYPE CORRELATION IN LYNCH SYNDROME

Although tumor predisposition is inherited with an autosomal dominant pattern, Lynch syndrome is a complex disease characterized by genetic heterogeneity and an extreme variability in phenotypic manifestations. Mutations in the MSH2 and MLH1 genes account for up to 70% of defects identified in Lynch families, while mutations in MSH6 and PMS2 are identified in the remaining 14% and 15% [68]. Differences in cancer risk, depending on gender and the mutated gene have been reported. The global risk associated with MSH6 and PMS2 mutations is substantially lower compared to the risk conferred by mutations in MLH1 and MSH2 [69]. 

Individuals with MSH2 mutations have a higher cumulative lifetime risk of developing primary extracolonic and cerebral tumors compared to MLH1 mutation carriers [70-72]. Different functions in the MMR system of MSH2 and MLH1 might be responsible for the variation in cancer risk observed. However, not only the mutated MMR gene but also the specific phenotype observed in the families should be taken into consideration while deciding the surveillance protocol [73]. In recent years, several founder/recurrent mutations in MMR genes have been identified which provide an important tool for population specific mutations [74]. The evaluation of a specific cancer risk attributable to these mutations may be helpful for clinical follow-up and patients’ management. For instance, a mutation in the MLH1 gene associated with a lower risk of endometrial and extracolonic cancers has been identified in the Danish population [75] while an MLH1 founder mutation associated with an aggressive phenotype and extracolonic manifestations has been described in Southern Italy [12]. Studies on the MSH2 A636P founder mutation in Ashkenazi Jews have shown a higher risk of CRC and endometrial cancer associated with this variant and led to the proposal of adopting a specific surveillance protocol in the mutation carriers [76]. The well known recurrent mutation in the MSH2 gene localized at intron 5 splice site, c.942+3A>T, and resulting in skipping of exon 5 [77,78], apparently causes a different gender specific cancer risk. Specifically, the risk of CRC and death associated with this mutation is higher in male carriers compared to females that instead present a significantly higher risk of ovarian cancer [79,80].

Patients harboring mutations in the MSH6 gene present a lower risk of CRC and a more advanced age at first tumor diagnosis [81,82]. It has been proposed that the presence of the MSH3 protein provides a partial compensation for the loss of MSH6 in the MMR functions [14]. In women carrying MSH6 mutations, the risk for endometrial cancer is doubled compared with that of MLH1 or MSH2 female mutation carriers [71]. Considering these features, for patients with a germline mutation in MSH6 a specific surveillance protocol has been proposed consisting of biennial colonoscopy starting by the age of 30 years instead of 20-25 years, while prophylactic hysterectomy should be considered from an age of 45 years [83]. The phenotypic variability and the frequent association with a MSI-L classification may complicate the identification of MSH6 mutation carriers [84].

PMS2 mutations have been identified in families meeting the RBG with tumors presenting isolated loss of PMS2 at IHC analysis [85]. The contribution of this gene to the LS pathogenesis has been overlooked until recent years for the difficulty to identify gene mutations due to the presence of pseudogenes. However, the use of PMS2-specific long range PCRs has increased the mutation detection rate [86,87]. The phenotypic consequence of PMS2 mutations seems to be highly variable, with weaker family history and older age of onset. The penetrance observed for PMS2 mutation carriers is lower compared with the one associated to mutations in other MMR genes but colorectal and endometrial cancer incidences are respectively 5 and 7-fold higher than in the general population [88]. 

Lynch syndrome also presents rare cases of biallelic mutations [89]. Both homozygosity and compound heterozygosity for MMR deficiency are not lethal while they cause early onset disease manifestations, which includes a neurofibromatosis-like phenotype (*cafè au lait* spots) often associated with leukemia or lymphoma, and cerebral and gastrointestinal malignancies [90].

Mutations in other genes of the MMR system, such as MLH3 and ExoI, have been rarely reported in association with familial CRC, but their association with LS is still debated [91,92]. These two genes have been taken into consideration since they are an integral part of MMR system but so far there are no compelling evidences of their role in LS pathogenesis.

An increasing number of LS cases caused by germline epigenetic inactivation of MLH1 has been reported [93-95]. These mutations, also referred as constitutional epimutations, have been identified in LS family with no mutations in MMR genes and early onset and/or multiple cancers [96,97]. A novel mechanism of MMR inactivation has been described in patients with IHC-negative MSH2 tumors, and tested negative in MSH2 mutational analysis. In these families germline deletions of the 3’ region of the EpCAM gene have been identified. MSH2 gene is localized just downstream to the EpCAM locus and deletions of this gene lead to MSH2 inactivation by two different mechanisms: i) methylation of the CpG island at 3’ of EpCAM gene which causes constitutional silencing of MSH2 [98], and ii) generation of an EpCAM-hMSH2 fusion transcripts [16]. Patients with EpCAM deletions present a high risk of CRC, while endometrial cancer risk apparently depends on the extension of the deleted region [99]. 

So far, over 1500 mutations in four major MMR genes have been described [100]. The International Society for Gastrointestinal Hereditary Tumors (InSIGHT) has established a database (www.insight-group.org) where all the identified variants and the results of functional and segregation studies eventually performed are listed [101]. The most common mutation type in the MSH2 gene is represented by large deletions (>30%) mediated by the presence of a cluster of Alu sequences [102]. Large rearrangements including deletions/duplications of single or multiple exons have been described also in MLH1 (5 %) [103-105]. 

The pathogenic role of mutations producing an unstable truncated protein is easily assessed since they cause lack of IHC staining in the tumor tissue. More difficult is the interpretation of the pathogenicity of missense mutations that account for 31% of MLH1 and 17% of MSH2 mutations [106]. Many different methods have been developed to evaluate the consequences on the protein function of these “Variants of Uncertain Significance” (VUS). In these cases, the first step in assessing their pathogenicity is to analyze the co-segregation of the variant within a family and the determination of its frequency in control populations. A second step is usually represented by the evaluation of the evolutionary conservation of these variants and/or of the region where they are localized. Essential domains for MMR proteins activity are the regions interacting with other MMR proteins or those with catalytic activity. In fact, missense mutations may directly affect protein function but can also interfere with protein stability, interactions or localization. Understanding how a single amino acid change contributes to disease pathogenesis is important for the management of mutation carriers harboring that particular variant. Both in silico and experimental procedures have been used in order to assess the pathogenicity of VUS [107], and a three-step model has been recently proposed [108,109]. Another bioinformatic algorithm based on evolutionary conserved sequences alignments, called Multivariate Analysis of Protein Polymorphisms-Mismatch Repair (MAPP-MMR), has been also developed to help in the pathogenetic classification of MMR-VUS [110]. Although functional studies are important to clarify the role of VUS [111], the loss of the repairing proficiency of the mutated protein by *in vivo* or *in vitro* assays is not easy to demonstrate [112]. 

The pathogenic role of some variants may also depend upon genetic background, environmental influences, life style, and modifier genes. All these factors have been hypothesized to explain the unanswered question of the high variability in cancer risk observed in LS patients, even among carriers harboring the same mutation. The understanding of the contribution of these factors, including low-risk alleles and modifier genes, in LS patients it is important to plan surveillance protocols tailored to the individual risk of cancer development. The majority of modifier genes investigated so far, have been selected on the basis of their putative biological role in cancer initiation or progression. This strategy has led to the identification of candidates such as the polymorphic CA repeat within the IGF1 promoter [113], a SNP within the xenobiotic metabolizing enzyme gene CYP1A1 [114] and many other candidate modifier genes that may profile the clinical presentation in MLH1 and MSH2 mutation carriers [115-118].

In few studies the quantitative evaluation of allele-specific expression of MMR genes in mutation carriers has been performed [119,120]. The allelic expression imbalance (AEI) is an additional factor that could contribute to variability in the disease phenotype [120]. Differential allelic expression can be influenced by sequence polymorphisms in regulatory regions as well as by the presence of mutations able to modify the level of gene expression. Analysis of AEI in NMD-insensitive mutation carriers revealed that MLH1 expression is influenced by two SNPs acting in *cis*, even if these influences are relatively small compared to variations in expression level caused by the presence of pathological mutations [121].

Recently, the introduction of genome-wide association studies (GWAS) revealed several SNPs that appear to influence CRC onset. Wijnen *et al. *[122] have identified two loci on 8q23.3 and 11q23.1 causing an increased risk of CRC in LS mutation carriers that were significantly associated with the number of risk alleles and an influence stronger in female carriers than in males. However, the role of these variants is still debated. A recent study on the French population did not find any association of these two loci with an increased CRC risk nor a sex specific risk [123], while a study on Australian and Polish populations has been able to replicate the association only in MLH1 mutation carriers [124]. The different results with previous studies may be due to genetic heterogeneity and different MMR mutations between analyzed populations and underline the necessity to increase the power of these studies to provide a definitive answer. 

## LYNCH SYNDROME AND ANTICIPATION

Genetic anticipation by Strachan and Read’s definition [125] is “a phenomenon in which the age of onset of a disorder is reduced and/or the severity of the phenotype is increased in successive generation”. For long time, geneticists have been skeptical on the real existence of the phenomenon. Clinical anticipation in diseases onset has been reported since 19^th^ century but it was for long time considered an ascertainment bias until the identification of the trinucleotide expansion mechanism in a group of inherited neuromuscular disorders. This mechanism represents the first molecular model able to explain the phenomenon of anticipation [126]. 

Today, genetic anticipation is a clinical feature characterizing a consistent number of neurodegenerative disorders known as dynamic diseases, which include, among the others, myotonic dystrophy, X-linked mental retardation, Huntington’s chorea, and Friedreich ataxia. The well-known molecular mechanism behind the progressively earlier onset and the increased severity observed in affected families is the generational expansion of trinucleotide repeats during successive meiosis. A sex-specific inheritance pattern has been proposed for the juvenile forms of these diseases because the triplets’ expansion occurs most frequently during male gametogenesis [127]. Evidence for genetic anticipation has been described also in cancer genetic syndromes like breast cancer, pancreatic cancer, ovarian cancer, leukemia, lymphoma and melanoma [128-133].

The progressive decrease of age at diagnosis of CRC in successive generation has been reported since the first description of Lynch syndrome in 1925 by Alfred Whartin [134]. Over the years many studies investigated on the possibility of anticipation in this syndrome (see Table **1**).

Menko and te Meerman reported the first clinical observation of anticipation in LS in 1993, but their data derived from families with uncertain mutation status and no statistical analysis was performed [135]. 

Vasen *et al. *[136] in a clinical study investigated the age of onset of CRC in 49 families with at least three relatives affected in two successive generations. In 41 families satisfying this minimum criterion for LS, a progressively earlier age of diagnosis of CRC in successive generations was observed. The remaining eight families presented later age of onset and the absence of extracolonic cancers frequently associated with LS. No molecular information was available for the families included in this study, and the author did not exclude the possibility of an ascertainment bias. 

In 1996, Rodriguez-Bigas *et al. *analyzed 40 families fulfilling LS criteria and confirmed the presence of a generational anticipation in the disease onset [137]. 

Fifty-one Lynch families selected according to the Amsterdam Criteria were analyzed in a statistical study by Voskuil *et al. *[138] who concluded that the earlier age of onset in successive generation does not appear to be a biological event, but reflects a secular time trend in cancer occurrence in the families included in the study.

The first study arguing against the existence of anticipation in familial colorectal cancer of unknown causes was published by Tsai *et al. *[139]. In this study 475 parent-child pairs (PCPs) with familial colorectal cancer were analyzed using statistical approach to correct for selection biases and cohort effects (many statistical tests were used including Gehan’s generalized Wilcoxon test, unpaired t-test, paired t-test, Pearson’s correlation coefficient, ANOVA). The authors found no evidence for an effect of parental gender on age at diagnosis in offspring, neither a secular trend toward younger onset of cancer. Tsai concluded that the clinical evidences of anticipation in LS previously reported probably reflected a birth cohort bias of ascertainment. MMR gene mutations status was known for only 14 of the 67 PCPs that met criteria for LS subgroup in this study. No difference in age at diagnosis between generations was observed in these families. Noteworthy, the mean age of parents and offspring was a decade younger in sporadic patients than in the LS subsample without identified mutations and the author could not exclude an association between MMR gene mutation and a very young onset of cancer.

The first evidence for genetic anticipation described presented some limitations: the limited power of statistical tests applied, the missing or incomplete molecular characterization of patients, and the different time windows analyzed. As the molecular-testing of Lynch patients became routinely performed most of the published studies reported the presence of genetic anticipation in the Lynch families analyzed.

Westphalen *et al. *[140] published a study on 55 affected parent-child pairs from 21 Swiss families with germline mutations in MLH1 (15) and MHS2 (6) genes. After the exclusion of a birth cohort effect, they observed 8 years anticipation, on average, in the age at tumor diagnosis in children compared with their parents. In particular, mutations in MLH1 were significantly associated with a more pronounced anticipation effect compared with MSH2 mutations, though this latter finding could have been due to the small sample size investigated. Furthermore, genetic anticipation appeared to be more evident when the mutation was paternally inherited.

In 2007 Stella *et al*. [141] first reported evidence of anticipation effects also in Lynch families segregating MSH2 mutations. The authors analyzed 24 affected parent-child pairs from four families with deletions in MSH2 and observed a median anticipation effect of 12 years. The authors found no indication of a birth cohort effect. It was not possible to evaluate the effect of the gender of the transmitting parent since only four of the PCP’s involved maternal transmission. In this work, PCPs were more numerous than the MSH2 PCPs analyzed by Westphalen (24 *vs* 14) and more homogeneous in terms of mutation type. Moreover, the analysis was not limited to PCPs in which both parents and offsprings developed CRCs including pairs where either the parents or the children were asymptomatic. MSH2 mutations are more likely to be associated with extracolonic tumors and nine of the 24 PCPs presented a tumor of this type. However, a significant anticipation also emerged restricting the analysis to the 14 PCPs in which both the members had developed CRCs as the first diagnosed cancer. It seems unlikely that the observed anticipation was significantly related to changes in surveillance policies because of the limited time distribution of patients. Still, it is possible that other genetic or environmental factors could play a role in the differences in age at cancer diagnosis of patients, although the influence of environmental factors is difficult to assess considering the variety of organs involved in Lynch syndrome. 

In 2008 a large Danish study on 290 parent-child Lynch pairs was published by Nilbert *et al. *showing a significant anticipation effect [142]. In this report, the children group developed a Lynch syndrome-associated cancer 9.8 years earlier, on average, compared to their relatives. They also analyzed different birth cohorts, all demonstrating anticipation including a 7.2 years earlier tumor development in the oldest cohort. To avoid biases linked to the variable follow-up length in different generations and to the presence of shared genetic and/or environmental factors the authors used a statistical method developed by Huang and Vieland [143]. Taking into consideration the variable length of the follow-up they were still able to demonstrate an anticipation effect of 5.5 years on average. Thus, two alternative statistical methods used in this work showed comparable results and the anticipation effect remained even when analyzing different birth cohorts, different parent-of-origin of mutation, different MMR gene mutated, and also after the exclusion of cancers developed during the follow up period. In the following year, the same authors [144] developed a parametric statistical model to test anticipation that was applied to a larger version (824 individuals from 125 families) of the Danish Lynch syndrome cohort they analyzed in their previous work. This model allowed the analysis of right-censored data, considered several generations and included unaffected family members. The inclusion of covariates is a substantial improvement and facilitates subgroup comparison and the analysis of possible confounders. A significant effect from anticipation was still evident in the studied cohort using this new statistical model.

A review of the statistical methods used for testing genetic anticipation has been published in 2010 by Boonstra *et al. *[145]. The authors applied the most common statistical tests to the Danish HNPCC-register and all the tests confirmed the presence of genetic anticipation in Lynch syndrome families. 

In summary, with the only exception of the study published by Tsai *et al. *[139], all the recent literature has reported evidences in favor of the presence of genetic anticipation in Lynch syndrome. The clinical observation of anticipation in LS families and the statistical analyses performed in different studies do not necessarily demonstrate the biological existence of this phenomenon. Until a molecular mechanism capable of explaining the genetic anticipation in LS will not be found, it’s not possible to rule out that the observed effects can be due to sampling errors or to the genetic and/or environmental common background of families. 

## MOLECULAR BASIS OF ANTICIPATION IN LYNCH SYNDROME

Similarly with the molecular mechanisms responsible for anticipation in dynamic diseases, mutations in MMR genes have been hypothesized to lead to the same type of generational instability in repetitive sequences of DNA and that this could represent the biological basis of genetic anticipation in Lynch syndrome.

In 1994, Shibata *et al*. [146] were the first to suggest that the younger onset of cancer observed in consecutive generations of Lynch families could be explained by the accumulation of mismatch repair slippage events due to the diminished DNA mismatch repair proficiency. Few years later, the same group using a mathematical model hypothesized that the number of mutations accumulated in a tumor was dependent on the mutation rate and the number of cell division [147].

The first evidence that mutations in microsatellite loci are present also in normal human tissue dates back to 2001 [148]. However, the normal tissue analyzed in this study derived from a single patient with homozygous deficiency of MLH1. 

Two different works published in 2005 have demonstrated the presence of low level of microsatellite instability in the peripheral blood lymphocytes (PBLs) of Lynch carriers long before tumor diagnosis [149,150]. Coolbaugh-Murphy *et al. *[150] observed an age-dependent increase of the instability in carriers PBLs compared with normal controls. In contrast, the level of MSI observed in PBLs DNA of patients with sporadic, MSI-H CRCs did not show any increase when compared with age-matched controls. The authors concluded that MSI is not due to circulating cells from MSI-H tumors. 

It is possible that germline mutations can already impair MMR proficiency prior to loss of heterozygosity partly contributing to early-onset cancers. The mutation carriers would carry this milder impairment since birth, slowly accumulating alterations in any replicating cell type until they reach levels associated with an appreciably increased cancer risk only later in life. 

If this is the case, the mutational load would be also present in carriers’ germ cells during the reproductive years leading to the transmission of an overloaded gamete to the next generations. As a consequence, the offspring would inherit a more pronounced risk of cancer development. The increase in the accumulated mutation burden in successive generations would determine a progressive earlier cancer onset until the mutation levels will preclude further transmission (e.g. due to childhood or intrauterine death or even absent gametogenesis). This hypothesis is supported by the evidence that when anticipation has been observed in inherited cancers, it is often limited to three consecutive generations [128]. Stella *et al. *[141] recently described an LS family where a female infant died of a ureter cancer at the age of 12 months. This case might be the result of the transmission of a particularly heavy mutational load, considered that the mutation was paternally inherited by a subject diagnosed with CRC at age 34.

So far only one study has investigated the association of MMR haploinsufficiency with a higher frequency of mutant microsatellite sequences in phenotypically normal cells [151]. In this study, a highly specialized and labor-intensive technique has been used to detect mutant microsatellite fragments in PBLs DNA that would be otherwise obscured by progenitor alleles when using traditional PCR-based genotyping. In all the seven patients analyzed in the study, there was a significantly higher frequency of mutant alleles for all the three loci tested compared to age-matched sporadic CRC patients and normal controls. The authors concluded that their findings might represent the first molecular mechanism able to explain anticipation effects in LS. The low levels of genetic instability observed in normal cells from MMR mutation carriers, if present also in the germline, would cause mutations accumulation and transmission of mutant alleles to the offspring at a significantly higher frequency than in non-carrier individuals. 

Considering the large number of repeat-containing genes that have been linked to the pathogenesis of these tumors, differences in the mutation frequency at these target loci would be identified in the offspring germline DNA only by massive screening approaches. As the development of a cancer requires multiple mutation steps [152], any single transmitted mutation would spare few years in this cascade, leading to the observed genetic anticipation in LS. 

Future investigations with more sensitive methods of MSI quantification in large series of Lynch carriers should be able to proof this hypothesis. 

## THE ROLE OF TELOMERE LENGTH IN ANTICIPATION

Telomere length attrition has been proposed as a novel mechanism of anticipation in different inherited diseases such as dyskeratosis congenita (DKC) [153] and Li-Fraumeni syndrome [154, 155]. In DKC, the telomerase haploinsufficiency, caused by mutations in the hTERC gene, which encodes for its catalytic subunit, causes a progressive telomere shortening in successive generations with an increased predisposition to malignancy associated to the inherited telomere length [156]. DKC represents a unique example of a direct interaction of the mutated gene with telomere length maintenance mechanisms. 

In Li-Fraumeni syndrome patients, carriers of germline mutations in the TP53 gene have shown an accelerated attrition of telomere length compared with both normal controls [157] and asymptomatic carriers [155]. Since p53 directly binds to the telomeric t-loop junctions and controls the cellular response to DNA damage, mutations in TP53 would allow cells to escape apoptosis and proliferate even in presence of critically short telomeres thus leading to a gradual heritable telomere shortening. Shorter telomeres in the germline of mutation carriers and their transmission to successive generations, combined with a faster lifetime attrition, may cause carriers to decrease telomere length under a critical threshold earlier in life triggering anticipated cancer onset [155]. A similar explanation has been proposed for patients with familial papillary thyroid cancer (FPTC) in which a shorter telomere length in PBLs has been observed compared with sporadic cases [158].

Both genetic anticipation and telomeres shorter than in normal age-matched controls have been observed also in familial breast cancer [159,160].

Bozzao *et al. *[161] recently reported the evaluation of the telomere length in patients with Lynch syndrome. The authors analyzed 38 MLH1 and 36 MSH2 mutation carriers from 21 Lynch families: 43 presented with a cancer of the LS spectrum, while 31 were asymptomatic. In this study, the mean telomere length in Lynch carriers did not differ from controls and declined with age in a similar manner. Conversely, affected patients demonstrated accelerated telomere attrition compared with normal individuals, whereas asymptomatic carriers had a slower decline with age.

MSH2 mutation carriers had significantly shortened telomeres compared with controls, whereas, in MLH1 carriers, the correlation between telomere length and age was loose and not significant. Furthermore, telomere attrition was correlated significantly with the age at onset in MSH2 carriers. In fact, patients who developed tumor relatively early had significantly longer telomeres than those who became symptomatic later in life. In contrast, MLH1 patients with advancing age of onset presented longer telomeres and increasing telomere length. These findings have been confirmed by a very recent study showing that MSH2 down-regulation in normal human fibroblasts induces an accelerated telomere shortening rate [162]. In MSH2^-/-^Terc^-/-^ mice, MSH2 deficiency abolished the anti-oncogenic effects of short telomeres in a p21/p53-dependent manner [163]. Haploinsufficiency for MSH2 gene may induce tolerance to very short telomeres, leading to faster telomere attrition, while a reduction in the MLH1 dosage could trigger homologous recombination-dependent ALT (Alternative Lengthening of Telomeres), causing telomere elongation. In fact, in human cells down-regulation of MLH1, but not of MSH2, lead to enhanced homologous recombination (HR), which is commonly believed to play a crucial role in ALT [164,165]. This model would explain the lack of a correlation between telomere length and age, which was observed only in MLH1 mutation carriers. The authors speculated that haploinsufficiency in somatic and germ cells of Lynch patients may slowly exert different effects on the telomere length inherited at birth, according to the presence of either MLH1 or MSH2 mutations. Thus, MSH2 and MLH1 genes may contribute differently to the balance between telomere shortening and lengthening activities according to the mechanisms depicted in Fig. (**1**).

## CONCLUSIONS

Mounting evidences show that anticipation is commonly associated with LS. The earlier onset of cancer in successive generations raises the issue of a stricter surveillance protocols in younger generations. It is likely that the telomere length may represent one of the factors that influences cancer onset and may also contribute to anticipation effects. However, other genetic and environmental factors could act in modifying cancer risk in Lynch syndrome patients. The identification of these additional factors may lead in a near future to a more precise definition of individual cancer risk and tailored clinical surveillance.

## Figures and Tables

**Fig. (1):**
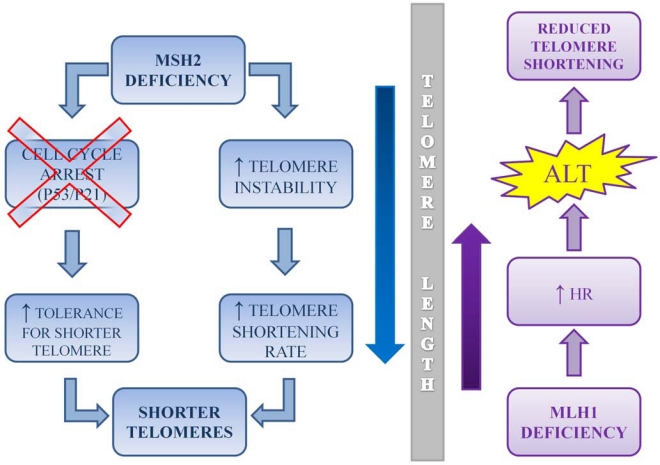
**A diagram illustrating telomere dynamics in MMR mutation carriers.** MSH2 deficiency leads to increased tolerance for shorter telomeres and telomere instability that accelerate telomere shortening. In a mouse Terc^-/-^ model this enhanced tolerance has been demonstrated to be p21/p53 dependent. In contrast, MLH1 deficiency causes an increase in homologous recombination (HR) which is considered one of the principle mechanisms for the alternative lengthening of telomeres (ALT) thus opposing to telomere shortening.

**Table 1:** Anticipation Studies in Lynch Syndrome

Author [Reference]	No. of analyzed patients (No. of asymptomatic carriers)	No. of analyzed families (No. with identified mutation)	No. of Analyzed PCPs	Average anticipation in years	No. of families with MMR genes mutations	Median age at onset according to mutated gene	Average anticipation in years according to the mutated gene	Statistical test used

Vasen [126]	194 (0)	41 (0)	ND	8.5 yrs **	ND	ND	ND	Paired t-test

Rodriguez-Bigas [127]	301 (17)	40 (0)	ND	YES (NA)	ND	ND	ND	NA

Voskuil [128]	1186 (956)	51 (12)	ND	NO	NA	ND	ND	Cox proportional hazards regression modelling

Tsai [129]	NA	38 (7)	67	NO	4 MLH1	ND	ND	Gehans generalized Wilcoxon test
					3 MSH2	ND	ND	

Westphalen [130]	83 (0)	21 (21)	55	8 yrs **	15 MLH1	42.5 (18-62) MLH1	11 yrs **	Wilcoxon matched pairs signed-ranks test
					6 MSH2	46 (29-60) MSH2	2 yrs NS	

Stella [131]	NA	4 (4)	24	12 yrs **	4 MSH2	-	-	Wilcoxon matched pairs signed-ranks test

Nilbert [132]	407 (0)	92 (92)	290	9.8 yrs **/5.5 yrs **	32 MLH1	46.7 (5-82) MLH1	10.10 yrs** / 5.42 yrs * MLH1	Paired t-test/Huang and Vielands bivariate model
					43 MSH2	44.4 (14-86) MSH2	7.58 yrs ** / 5.11 yrs ** MSH2	
					17 MSH6	52.9 (18-83) MSH6	9.75 yrs **/ 6.43 yrs * MSH6	

Larsen [134]	824 (0)	125 (125)	NA	3 yrs **	44 MLH1	ND	2.86 yrs # MLH1	Parametric model
					59 MSH2	ND	2.49 yrs # MSH2	
					22 MSH6	ND	5.13 yrs # MSH6	

ND= Not Done; NA=Not Available; NS= Not Significant.

* p<0.05,

** p<0.001,

# p value not applicable.
